# Supplementary material for: Mesophotic benthic communities associated with a submerged palaeoshoreline in Western Australia
Source: PLoS One. 2023 Aug 16;18(8):e0289805. doi: 10.1371/journal.pone.0289805 (PMC10431660; doi:10.1371/journal.pone.0289805)
Supplement: S1 Fig — (PDF) [file pone.0289805.s001.pdf]

S1 Fig. Methods description for Sediment analysis undertaken by Geoscience Australia.

Methods

The aim of this study was to find, if present, indicators of a submerged shoreline from sediment recovered from the seabed. The best-case scenario would be to find sediment particles indicative of a shoreline that were originally in situ at the time of collection, and not transported. Examples would include articulated intertidal/estuarine bivalves, and/or mangrove or other plant remains, or perhaps reddish (i.e., terrestrial) sediment. These were therefore the targets of this study.

Subsamples of sediment retained at Geoscience Australia and collected by the AIMS vessel R. V. Solander, were obtained from those samples submitted for grain size analysis. Approximately 50-150 ml of sediment was taken from each original sample for this sediment analysis after homogenization. The retrieved subsamples were wet sieved over a 63 um sieve, the mud fraction (< 63 um) not retained, and the sample air dried. Each sample was then lightly dry sieved over 2000 um and a 250 um sieves to enable observations on the gravel and medium to coarse sand. Proportions of identifiable skeletal carbonate particles were determined using standard percentage area diagrams (Figure 1).

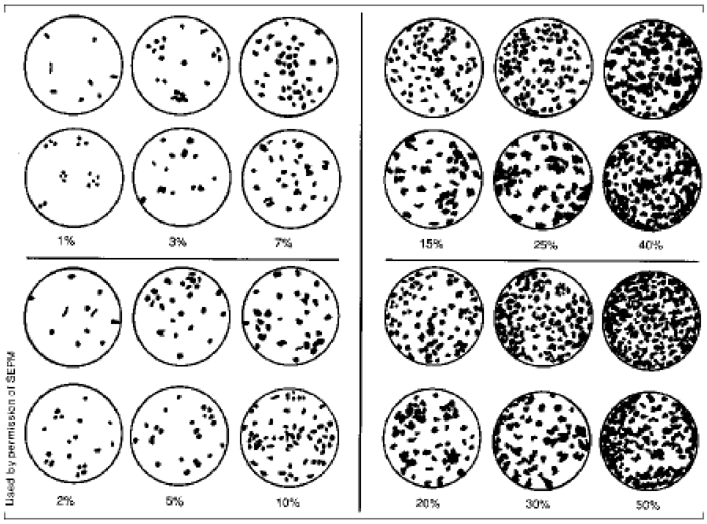

Fig 1. Percentage area chart used in this study.

The contents of each aliquot were visually assessed to be Modern, Relict, or Mixed, based on the proportions of well preserved, and poorly preserved skeletal carbonate, and to a lesser extent on the preservation state of the non-skeletal carbonate component. This was done without reference to a sample's depth, nor location on the shelf, effectively a 'blind' analysis. Modern refers to sediment where particles are largely unbroken, retain

original colour, are otherwise not discoloured, and do not have any secondary cementation. Relict refer to particles that are predominantly lacking in original colour or are discoloured to browns or greys/black, fractured and/or broken, and generally secondarily cemented. Mixed sediments refer to mixed proportions in a single sample.

Sediment grain size was determined using standard sieves and laser diffraction techniques, the latter using a Malvern Mastersizer 3000 at the laboratories of Geoscience Australia. Based on observations that sieve and laser methods give equivalent results using spherical particles, the laser and sieve results were combined to produce grain size distributions for the range fine Clay to very coarse Gravel. From the available grain size data for these sediments' values for Mud %, Sand %, Gravel %, Folk and Ward (1957) Mean grain size was determined. Sediment textures were calculated for the Folk (1980) textures for each sample of sediment. The results are presented for each sample on the following graphs, as well as observations on geomorphic features present.

References

Folk RL, Ward WC. A Study in the Significance of Grain-Size Parameters. J. Journal of Sedimentary Petrology. 1957; 27, 3-26.

Folk RL. Petrology of Sedimentary Rocks. Hemphill Publishing Company. 1980; Austin, 184 p.

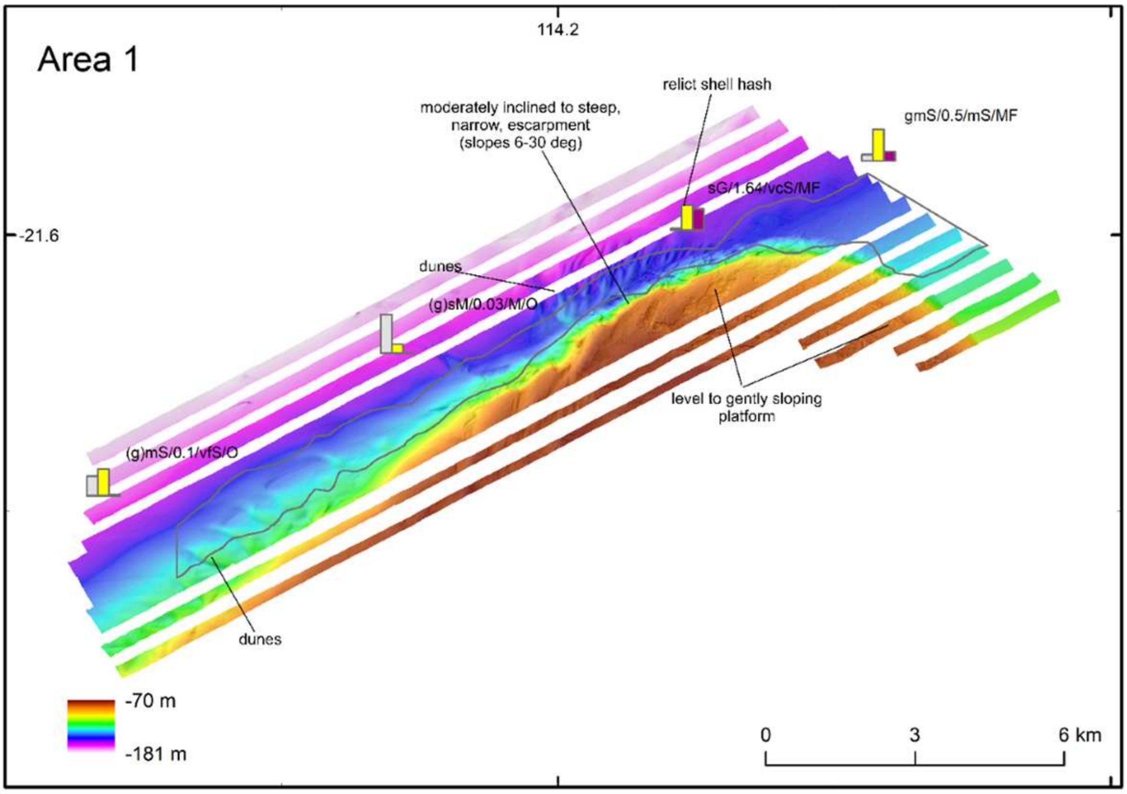
Area 1
114.2
-21.6
moderately inclined to steep, narrow, escarpment (slopes 6-30 deg)
relict shell hash
gmS/0.5/mS/MF
sG/1.64/vcS/MF
dunes
(g)sM/0.03/M/O
level to gently sloping platform
(g)mS/0.1/vfS/O
dunes
-70 m
-181 m
0
3
6 km

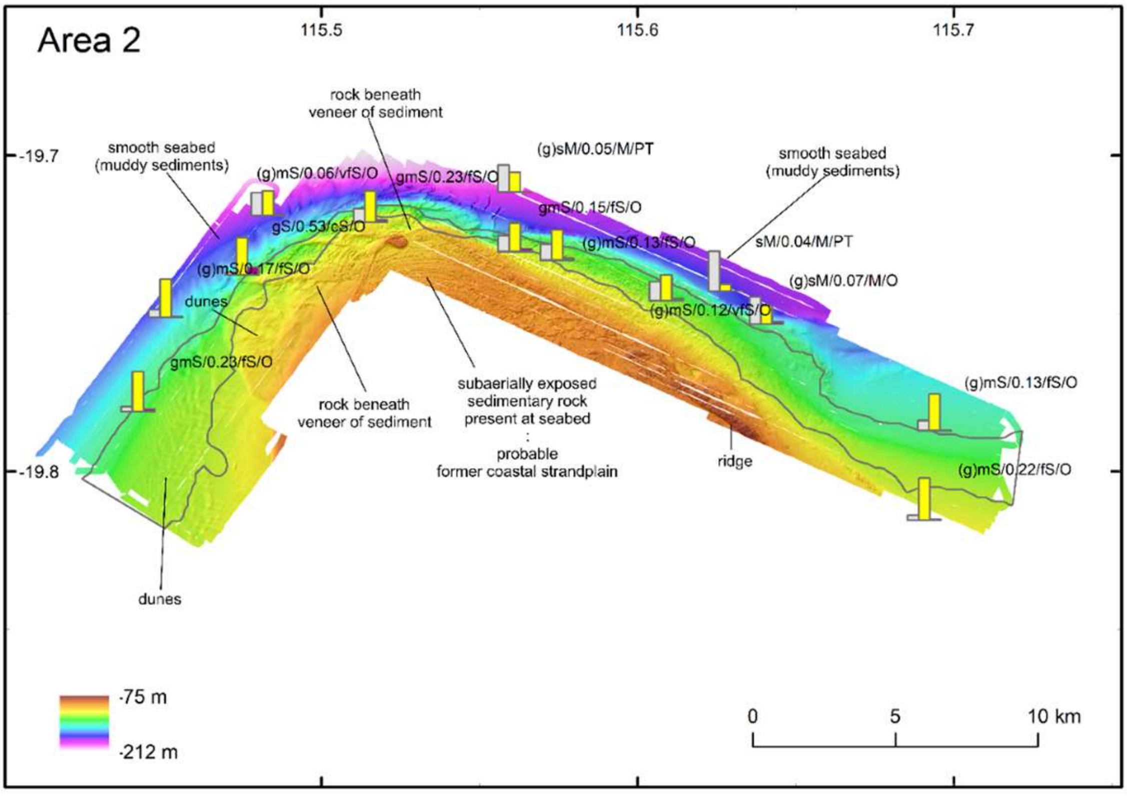
Area 2
115.5
115.6
115.7
-19.7
-19.8
rock beneath veneer of sediment
smooth seabed (muddy sediments)
(g)mS/0.06/vfS/O
gmS/0.23/fS/O
(g)sM/0.05/M/PT
smooth seabed (muddy sediments)
gmS/0.15/fS/O
gS/0.53/cS/O
(g)mS/0.13/fS/O
sM/0.04/M/PT
(g)mS/0.17/fS/O
(g)sM/0.07/M/O
dunes
(g)mS/0.12/vfS/O
gmS/0.23/fS/O
subaerially exposed sedimentary rock present at seabed
:
probable former coastal strandplain
(g)mS/0.13/fS/O
rock beneath veneer of sediment
ridge
(g)mS/0.22/fS/O
dunes
-75 m
-212 m
0
5
10 km

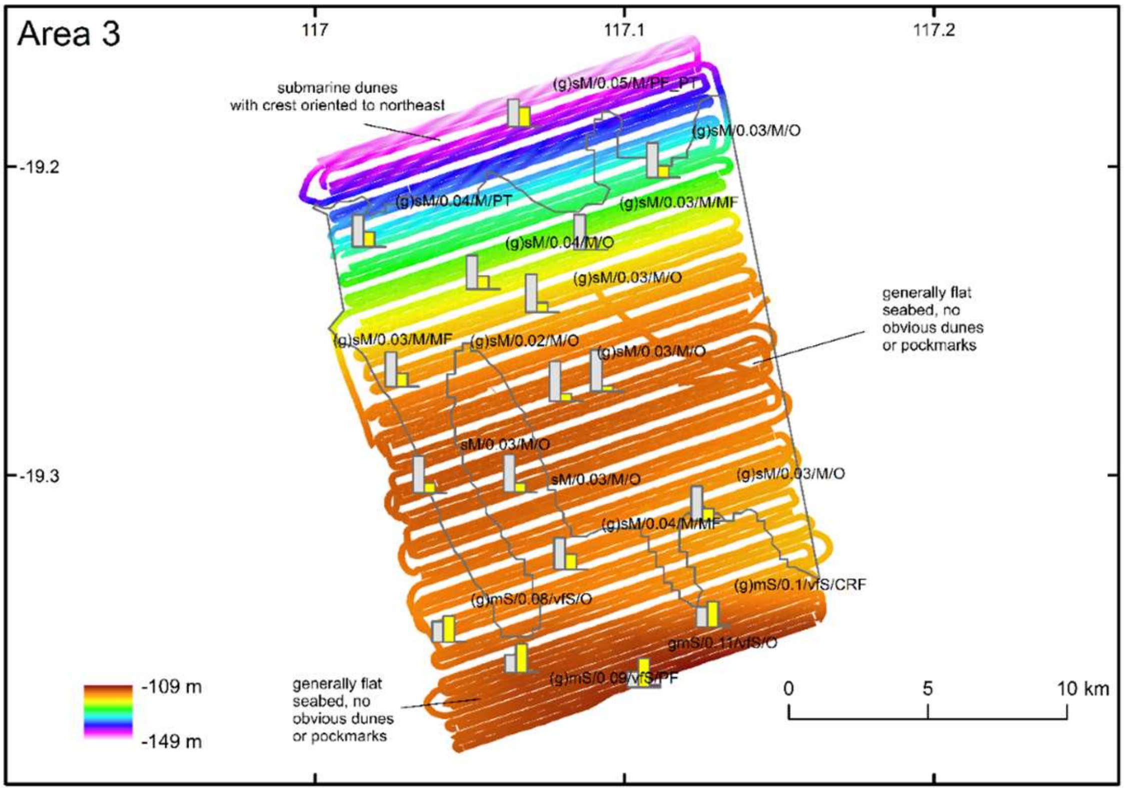
Area 3
117
117.1
117.2
-19.2
-19.3
submarine dunes with crest oriented to northeast
(g)sM/0.05/M/PF_PT
(g)sM/0.03/M/O
(g)sM/0.04/M/PT
(g)sM/0.03/M/MF
(g)sM/0.04/M/O
(g)sM/0.03/M/O
generally flat seabed, no obvious dunes or pockmarks
(g)sM/0.03/M/MF
(g)sM/0.02/M/O
(g)sM/0.03/M/O
sM/0.03/M/O
sM/0.03/M/O
(g)sM/0.03/M/O
(g)sM/0.04/M/MF
(g)mS/0.1/vfS/CRF
(g)mS/0.08/vfS/O
gmS/0.11/vfS/O
(g)mS/0.09/vfS/PF
-109 m
-149 m
generally flat seabed, no obvious dunes or pockmarks
0
5
10 km

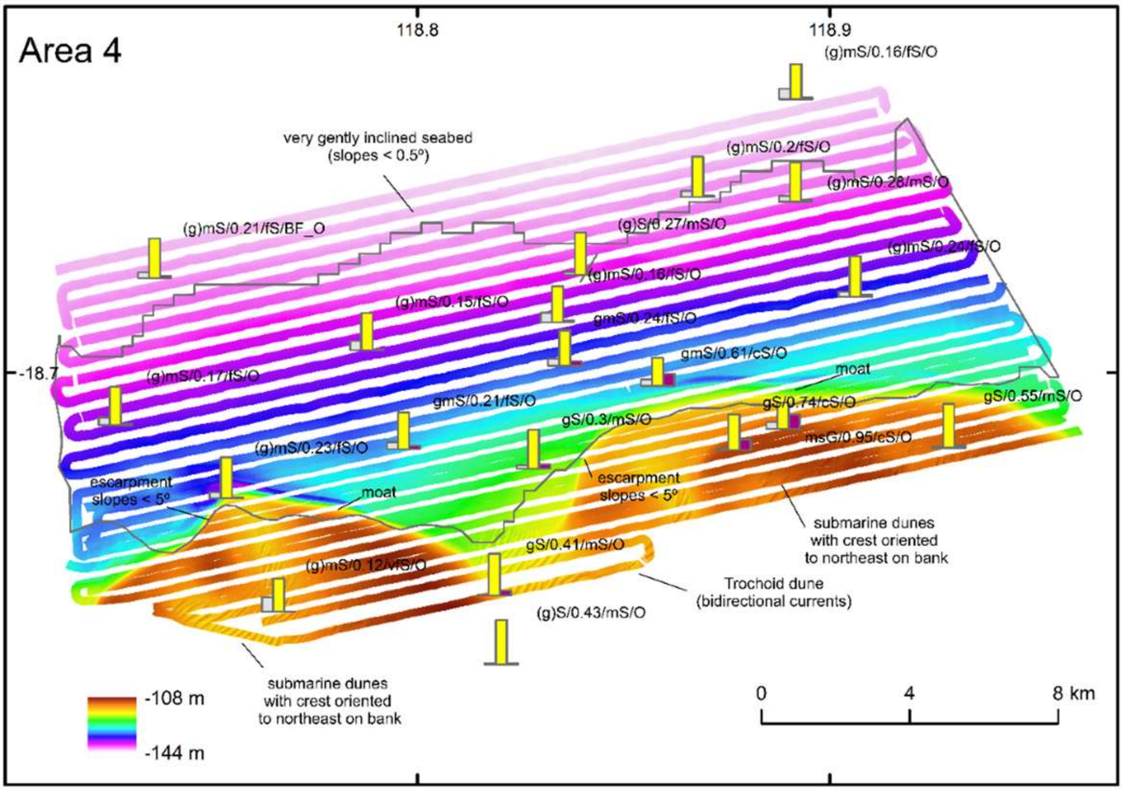
Area 4
118.8
118.9
-18.7
(g)mS/0.16/fS/O
very gently inclined seabed (slopes < 0.5º)
(g)mS/0.2/fS/O
(g)mS/0.28/mS/O
(g)mS/0.21/fS/BF_O
(g)S/0.27/mS/O
(g)mS/0.24/fS/O
(g)mS/0.16/fS/O
(g)mS/0.15/fS/O
gmS/0.24/fS/O
gmS/0.61/cS/O
(g)mS/0.17/fS/O
moat
gS/0.74/cS/O
gS/0.55/mS/O
gmS/0.21/fS/O
gS/0.3/mS/O
msG/0.95/cS/O
(g)mS/0.23/fS/O
escarpment slopes < 5º
moat
escarpment slopes < 5º
submarine dunes with crest oriented to northeast on bank
gS/0.41/mS/O
(g)mS/0.12/vfS/O
Trochoid dune (bidirectional currents)
(g)S/0.43/mS/O
submarine dunes with crest oriented to northeast on bank
-108 m
-144 m
0
4
8 km

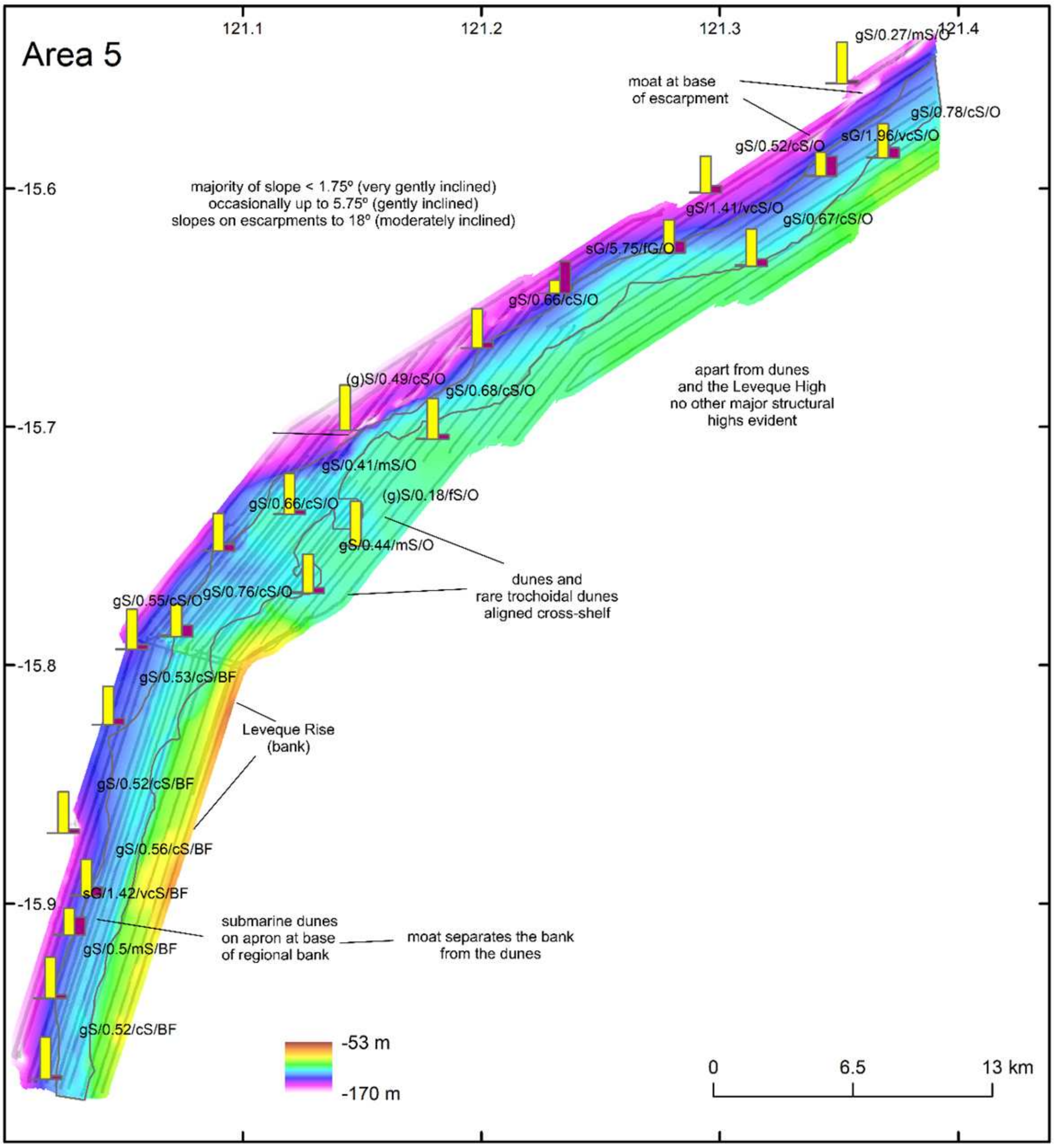

Sediment labels:
Sediment Texture (Folk) / grainsize (mm) / grainsize / principal skeletal carbonate components

mud %
sand %
gravel %
AC125 KEF

(g)mS:	slightly gravelly muddy Sand	cS:	coarse Sand	BF:	benthic foraminifers
(g)S:	slightly gravelly Sand	fG:	fine Gravel	BF_O:	benthich foraminifers, ooids
(g)sM:	slightly gravelly Mud	fS:	fine Sand	CRF:	carbonate rock fragments
gmS:	gravelly muddy Sand	M:	Mud	MF:	mollusc shells +/- fragments
gS:	gravelly Sand	mS:	muddy Sand	O:	ooids
msG:	muddy sandy Gravel	vcS:	very coarse Sand	PF:	planktonic foraminifers
sG:	sandy Gravel	vfS:	very fine Sand	PT:	pteropods
sM:	sandy Mud			PF_PT:	planktonic foraminifers, pteropods
