# Supplementary material for: Mesophotic benthic communities associated with a submerged palaeoshoreline in Western Australia
Source: PLoS One. 2023 Aug 16;18(8):e0289805. doi: 10.1371/journal.pone.0289805 (PMC10431660; doi:10.1371/journal.pone.0289805)

S3 Fig. Towed video transects at the five study areas. Maps show the AC125 (blue) and areas shallower (light gray) and deeper (dark gray) than the AC125. Transect lines are coloured to indicate benthic composition and bar plots show the biotic composition of transects. n = the total number of transects surveyed in each area. Only transects with biota are shown in bar graphs.

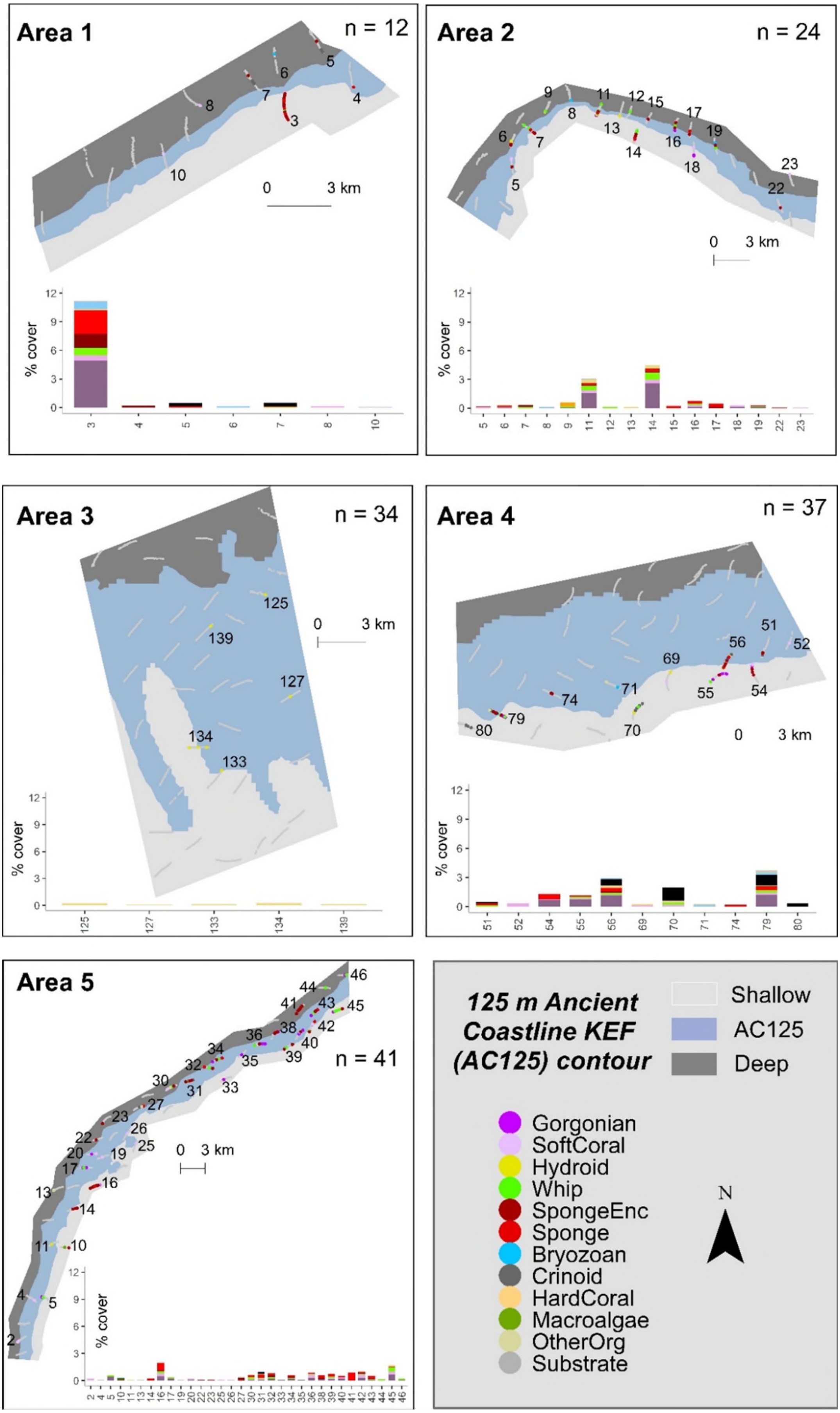

Supplement: S2 Fig — Maps show the AC125 (blue) and areas shallower (light gray) and deeper (dark gray) than the AC125. Transect lines are coloured to indicate benthic composition and bar plots show the biotic composition of transects. n = the total number of transects surveyed in each area. Only transects with biota are shown in bar graphs. (PDF) [file pone.0289805.s002.pdf]
