# Supplementary material for: Mesophotic benthic communities associated with a submerged palaeoshoreline in Western Australia
Source: PLoS One. 2023 Aug 16;18(8):e0289805. doi: 10.1371/journal.pone.0289805 (PMC10431660; doi:10.1371/journal.pone.0289805)

S4 Fig. Downward-pointing still imagery from towed video surveys showing a) example of microbenthos, b) filter feeder community, c) crinoids and d) hard coral colony.

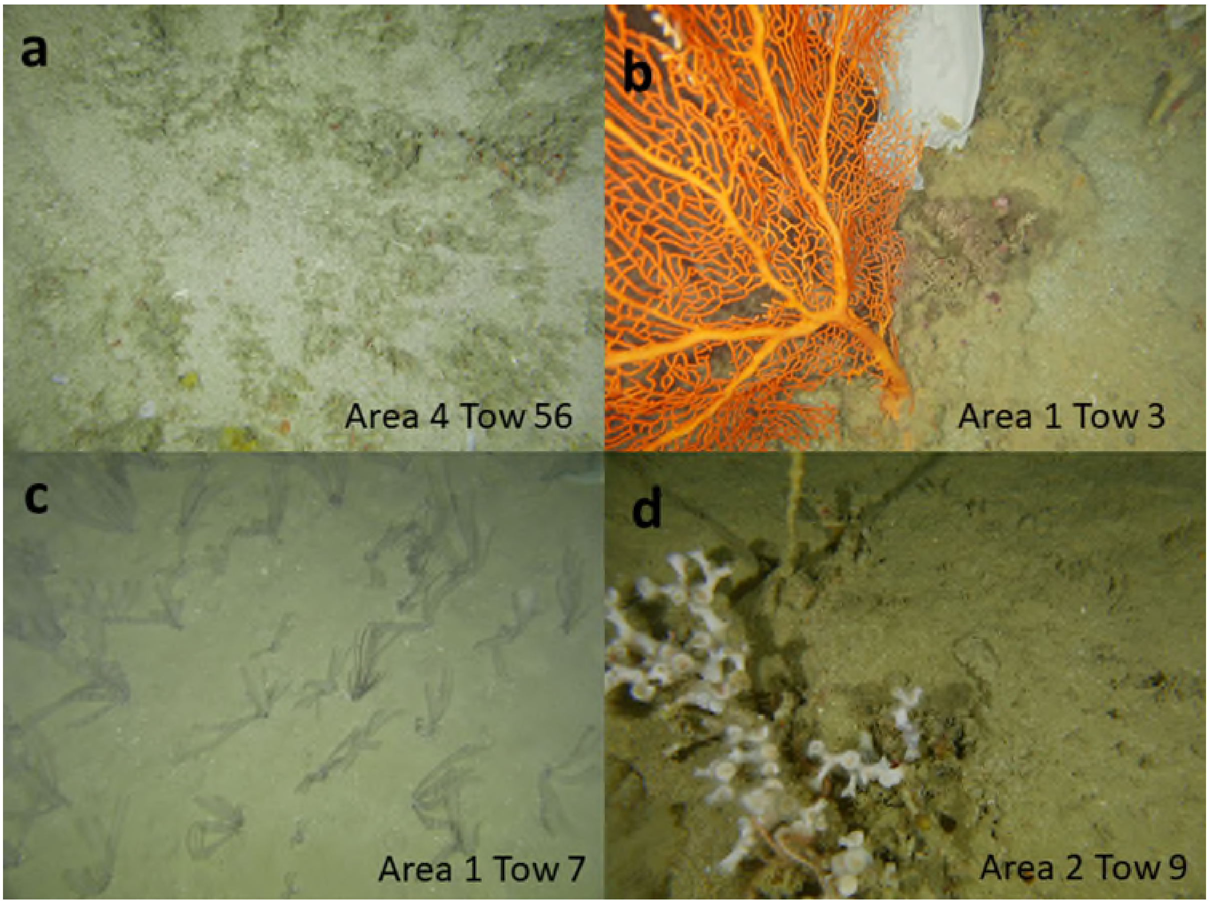

Supplement: S3 Fig — Downward-pointing still imagery from towed video surveys showing a) example of microbenthos, b) filter feeder community, c) crinoids and d) hard coral colony. (PDF) [file pone.0289805.s003.pdf]
