# Supplementary material for: Mesophotic benthic communities associated with a submerged palaeoshoreline in Western Australia
Source: PLoS One. 2023 Aug 16;18(8):e0289805. doi: 10.1371/journal.pone.0289805 (PMC10431660; doi:10.1371/journal.pone.0289805)
Supplement: S4 Fig — (PDF) [file pone.0289805.s004.pdf]

S6 Fig. Significant pairwise tests within the interactive term 'Area x Position' are coloured red (P ≤ 0.05), with non-significant tests coloured green.

Position Shallow

1					
2					
3	0.0110	0.0004			
4			0.0009		
5					
	1	2	3	4	5

Position AC125

1					
2					
3		0.0002			
4					
5			0.0010		
	1	2	3	4	5

Position Deep

1					
2					
3					
4		0.0066			
5					
	1	2	3	4	5

Area 1

Shallow			
AC125			
Deep			
	Shallow	AC125	Deep

Area 2

Shallow			
AC125	0.0311		
Deep	0.0066		
	Shallow	AC125	Deep

Area 3

Shallow			
AC125			
Deep			
	Shallow	AC125	Deep

Area 4

Shallow			
AC125			
Deep	0.024		
	Shallow	AC125	Deep

Area 5

Shallow			
AC125			
Deep			
	Shallow	AC125	Deep
