# Supplementary material for: Mesophotic benthic communities associated with a submerged palaeoshoreline in Western Australia
Source: PLoS One. 2023 Aug 16;18(8):e0289805. doi: 10.1371/journal.pone.0289805 (PMC10431660; doi:10.1371/journal.pone.0289805)

S8 Fig. Relative importance of predictor variables in the global benthic habitat model fit to the five areas (refer to Table 1 for explanation of variables). The y-axis represents mean value with error bars representing permuted importance for each variable using the Gini index value. The Gini index value n machine learning is a measure based on the sum of the error reduction aka reduced entropy when this variable is included in the random Forest models. It is also known as mean decrease in impurity (MDI).

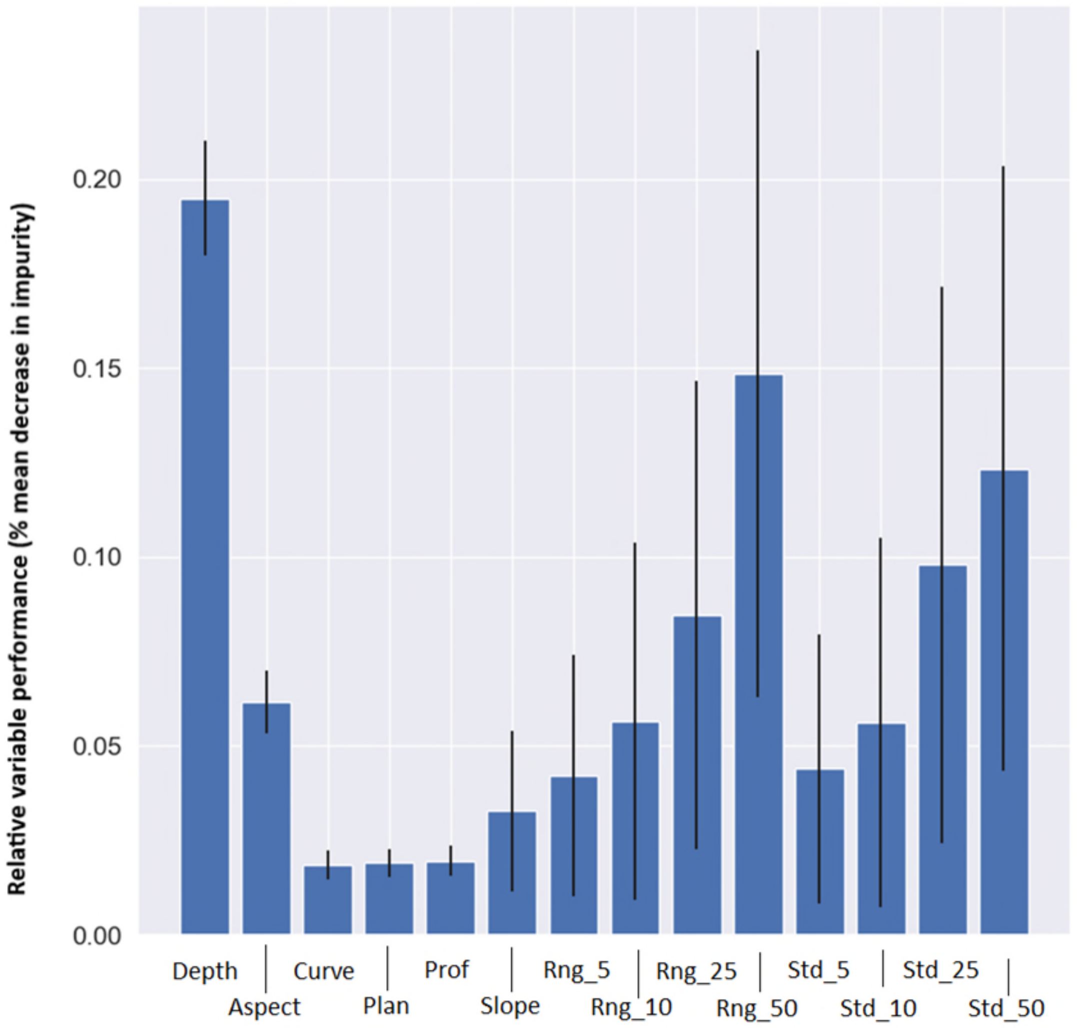

Supplement: S5 Fig — (PDF) [file pone.0289805.s005.pdf]
