# Supplementary material for: Mesophotic benthic communities associated with a submerged palaeoshoreline in Western Australia
Source: PLoS One. 2023 Aug 16;18(8):e0289805. doi: 10.1371/journal.pone.0289805 (PMC10431660; doi:10.1371/journal.pone.0289805)
Supplement: S1 Table — (PDF) [file pone.0289805.s006.pdf]

S2 Table. Percentage cover of benthic groups at the five study areas.

			Location				
	**Benthic group**	**Description**	Area1	Area2	Area3	Area4	Area5
Soft Coral	Gorgonian	fans, rigid soft corals	0.41	0.20		0.10	0.10
	Whip	c.f. *Junceella*	0.07	0.07		0.02	0.05
	Soft coral	all other soft corals including seapens and black coral	0.07	0.05		0.04	0.06
Sponge	Encrusting sponge	encrusting, creeping	0.23	0.07		0.05	0.11
	Sponge	erect, massive, cups	0.14	0.02		0.00	0.02
Other Biota	Crinoid	all crinoids	0.05			0.10	0.01
	Hydroid	all hydroids	0.01	0.04	0.02	0.02	0.01
	Bryozoan	all bryozoa	0.07	0.01		0.01	
	Other organisms	seastar, ascidian, anemone, urchin	0.02			0.01	0.01
	Hard coral	branching		0.02			
	Macroalgae	red algae					0.01
Subtotal biotic cover			***1.07***	***0.48***	***0.02***	***0.36***	***0.38***
Other Substrate	Microbenthos	abiotic/biotic on sand or rubble	0.12	0.16	0.14	0.97	0.02
	Soft substrate	sand/silt/mud	96.13	97.44	99.80	97.25	98.50
	Consolidated	cobble, boulder, bedrock	2.57	1.87	0.04	0.35	1.00
	Rubble	small rock/pebble; unconsolidated	0.12	0.05		0.08	0.11
