# Supplementary material for: Mesophotic benthic communities associated with a submerged palaeoshoreline in Western Australia
Source: PLoS One. 2023 Aug 16;18(8):e0289805. doi: 10.1371/journal.pone.0289805 (PMC10431660; doi:10.1371/journal.pone.0289805)
Supplement: S2 Table — (PDF) [file pone.0289805.s007.pdf]

S5 Table. Towed video transect data summarised by study area.

Area	Transects (n)	Images (n)	Points (n)	Depth min (m)	Depth max (m)	Depth mean (m)	Depth SE
1	12	2138	10,529	62	166	135	0.21
2	24	5651	28,170	93	204	140	0.12
3	34	5991	28,976	112	150	120	0.05
4	37	5650	27,132	105	139	125	0.06
5	41	8976	44,268	92	171	126	0.08
**Total**	**148**	**28,406**	**139,075**	**93**	**166**	**129**	**0.52**
