# Supplementary material for: Mesophotic benthic communities associated with a submerged palaeoshoreline in Western Australia
Source: PLoS One. 2023 Aug 16;18(8):e0289805. doi: 10.1371/journal.pone.0289805 (PMC10431660; doi:10.1371/journal.pone.0289805)
Supplement: S3 Table — (PDF) [file pone.0289805.s008.pdf]

S7 Table. Testing and training data distribution by study area and habitat class.

Habitat class	Testing and training data sample size in the study area					
	**Area 1**	**Area 2**	**Area 3**	**Area 4**	**Area 5**	**TOTAL**
Burrower	0	26	0	0	0	26
Filter feeders - dense	0	126	0	0	0	126
Filter feeders - medium	640	2162	2	782	2350	5936
Filter feeders - sparse	2318	5728	992	4844	16,169	30,051
Gorgonian - medium	19	0	0	0	0	19
Gorgonian - sparse	32	0	0	14	0	46
No biota detected	4934	8144	20,866	17,625	13,789	65,358
Soft Coral - medium	0	0	0	0	6	6
Soft Coral - sparse	0	0	0	1	1	2
Sponge - sparse	1	0	11	9	3	24
Whips - sparse	22	11	6	0	1	40
TOTAL	7966	16197	21,877	23,275	32,319	101,634
