# Supplementary material for: Mesophotic benthic communities associated with a submerged palaeoshoreline in Western Australia
Source: PLoS One. 2023 Aug 16;18(8):e0289805. doi: 10.1371/journal.pone.0289805 (PMC10431660; doi:10.1371/journal.pone.0289805)
Supplement: S4 Table — (PDF) [file pone.0289805.s009.pdf]

S9 Table. Percentage of three dominant habitat classes (plus a mixed class: Other biota) in each of five study areas.

**Habitat class**	**% study area**					
	**Area 1**	**Area 2**	**Area 3**	**Area 4**	**Area 5**	**Average**
No biota detected	39.03	48.52	99.79	83.25	48.19	64.64
Filter feeders - sparse	36.90	44.62	0.21	15.17	47.82	28.58
Filter feeders - medium	24.05	6.73	0.00	1.58	3.98	6.75
Other biota	0.01	0.13	7.52	0.00	0.01	0.03
**Classification accuracy (kappa)**	0.9724	0.9097	0.7618	0.9377	0.9219	N/A
