# Supplementary material for: Mesophotic benthic communities associated with a submerged palaeoshoreline in Western Australia
Source: PLoS One. 2023 Aug 16;18(8):e0289805. doi: 10.1371/journal.pone.0289805 (PMC10431660; doi:10.1371/journal.pone.0289805)
Supplement: Attachment — Submitted filename: Response to Reviewers.docx [file pone.0289805.s012.docx]

Response to Reviewers

Reviewer 1: I have incorporated and addressed your suggestions into my revision. They were very helpful. Thank you.

Reviewer 2: I have incorporated and addressed your suggestions into my revision. Thank you for your help.
